# Correlation between Commercial Anti-RBD IgG Titer and Neutralization Titer against SARS-CoV-2 Beta Variant

**DOI:** 10.3390/diagnostics11122216

**Published:** 2021-11-27

**Authors:** Rosana Wing-Shan Poon, Lu Lu, Carol Ho-Yan Fong, Tak-Chuen Ip, Lin-Lei Chen, Ricky Rui-Qi Zhang, Cyril Chik-Yan Yip, Vincent Chi-Chung Cheng, Kwok-Hung Chan, Kwok-Yung Yuen, Kelvin Kai-Wang To

**Affiliations:** 1 Department of Microbiology, Queen Mary Hospital, Hong Kong, China; rosana@hku.hk (R.W.-S.P.); chuen1220@gmail.com (T.-C.I.); cyrilyip2@gmail.com (C.C.-Y.Y.); vcccheng@hku.hk (V.C.-C.C.); kyyuen@hku.hk (K.-Y.Y.); 2 Department of Microbiology, Li Ka Shing Faculty of Medicine, The University of Hong Kong, Hong Kong, China; u3003963@connect.hku.hk (L.L.); carolfong.hy@gmail.com (C.H.-Y.F.); chenlinlei2346@gmail.com (L.-L.C.); zhangrq@hku.hk (R.R.-Q.Z.); chankh2@hku.hk (K.-H.C.); 3 State Key Laboratory for Emerging Infectious Diseases, Carol Yu Centre for Infection, Department of Microbiology, Li Ka Shing Faculty of Medicine, The University of Hong Kong, Pokfulam, Hong Kong, China

**Keywords:** COVID-19, SARS-CoV-2, antibody, receptor binding domain, neutralization

## Abstract

Objectives: The emergence of SARS-CoV-2 variants of concern (VOCs) have diminished the effectiveness of vaccines and are associated with a rebound in the number of COVID-19 cases globally. These variants contain mutations at the spike (S) protein receptor binding site (RBD), which affect antibody binding. Current commercially available antibody assays were developed before the VOCs emerged. It is unclear whether the levels of these commercially available antibody assays can predict the neutralizing antibody titers against the VOCs. In this study, we sought to determine the correlation between the binding antibody concentration and microneutralization antibody titer against the beta variant. Methods: This study included 58 COVID-19 patients. The concentrations of IgG against the SARS-CoV-2 spike protein RBD and nucleocapsid (N) protein were measured using the Abbott SARS-CoV-2 IgG II Quant assay and the SARS-CoV-2 IgG assay, respectively. The neutralization antibody titer against the wild type lineage A SARS-CoV-2 and against the beta variant (B.1.351) was determined using a conventional live virus neutralization test. Results: The geometric mean MN titer (GMT) against the beta variant was significantly lower than that against the wild type lineage A virus (5.6 vs. 47.3, *p* < 0.0001). The anti-RBD IgG had a better correlation with the neutralizing antibody titer than that of the anti-N IgG assay against the wild type lineage A virus (Spearman rho, 0.5901 vs. 0.3827). However, the correlation between the anti-RBD or the anti-N IgG and the MN titer against the beta variant was poor. Conclusions: Currently available commercial antibody assays may not predict the level of neutralizing antibodies against the variants. A new generation of antibody tests specific for variants are required.

## 1. Introduction

The COVID-19 pandemic has constituted a major threat to humans. Serological assays for SARS-CoV-2 have played important roles in clinical management, outbreak control, and serosurveillance studies [1,2]. For diagnostics, antibody tests are especially useful for patients whose molecular test results are inconclusive, if the patient presents late and the virus is already cleared, or if the patient has a suspected reinfection [3,4]. Seroprevalence studies are pivotal in uncovering the true burden of infection, as infections confirmed by RT-PCR only represent the tip of the iceberg [2]. Serological assays are also critical for evaluating the immunogenicity of vaccines, and this is especially important with the emergence of variants. During contact tracing, the combination of epidemiological and antibody test information can help to identify the source of an outbreak [4]. Antibody tests have helped to guide decisions on patient discharge, especially for patients with prolonged viral shedding.

There are three major types of serological assays for COVID-19 [1,5]. Antibodies against a specific SARS-CoV-2 protein, such as the N protein or the S protein, can be detected by a chemiluminescence immunoassay (CLIA), an enzyme immunoassay assay (EIA), or a lateral flow assay (LFA) [6]. Neutralizing antibodies can be measured by a conventional live virus neutralization test (cVNT), pseudovirus neutralization assay, and surrogate virus neutralization assay (sVNT) [7,8]. Although cVNT is the “gold standard” serological assay for diagnosis and determination of protective immunity, it can only be performed in biosafety level 3 laboratories and is time consuming. On the other hand, CLIA, EIA, LFA, or sVNT are technically easy and can be performed in biosafety level 2 laboratories, and these assays are available commercially.

Since late 2019, several SARS-CoV-2 variants with RBD mutations have emerged. The World Health Organization has classified the alpha (B.1.1.7), beta (B.1.351), gamma (P.1), and delta (B.1.617.2) variants as VOCs [9]. The beta, gamma, and delta variants are more resistant to neutralization by the convalescent serum collected from COVID-19 vaccine recipients or from patients naturally infected by SARS-CoV-2 without RBD mutations [10]. Since current commercial antibody assays were designed based on the wild type SARS-CoV-2, we hypothesized that the antibody concentration determined by these commercial assays may not correlate well with the neutralizing antibody titer. In this study, we sought to determine the correlation between the binding antibody concentration and microneutralization antibody titer against the beta variant.

## 2. Materials and Methods

### 2.1. Patient Specimens

A total of 58 archived serum specimens from COVID-19 patients were retrieved from the Clinical Microbiology Laboratory of Queen Mary Hospital. These specimens were collected between February and May 2020 at a median of 36 days post symptom onset (IQR: 23–46 days). These patients had not received a COVID-19 vaccine before the collection of the serum specimens. This study was approved by the Institutional Review Board of the University of Hong Kong/Hospital Authority Hong Kong West Cluster (UW 13–372). Written informed consent was waived as only archived specimens were used.

### 2.2. Chemiluminescent Microparticle Immunoassay for the Detection of Anti-N and Anti-RBD IgG

We tested specimens for anti-N IgG using the Abbott SARS-CoV-2 IgG assay and for anti-RBD IgG using the SARS-CoV-2 IgG II Quant assay according to the manufacturer’s instructions. Both assays, based on the CLIA platform, were performed on the Architect i2000 SR immunoassay analyzer (Abbott Diagnostics, Chicago, IL, USA).

The manufacturers’ recommended cut-off for seropositivity was a S/CO value of 1.4 for the anti-N IgG qualitative assay and 50 AU/mL for the anti-RBD IgG quantitative assay.

### 2.3. Conventional Live Virus Neutralization Assay

A cVNT assay was performed as we described previously [10,11]. The virus isolates included the SARS-CoV-2 HKU-001a (wild type lineage A; GenBank accession number MT230904) [12] and the beta variant (Lineage B.1.351; GISAID accession number: EPI_ISL_2423556). The microneutralization (MN) antibody titer was the highest dilution with a 50% inhibition of cytopathic effect. An MN titer of ≥10 was considered positive. A viral culture of SARS-CoV-2 and cVNT assays were conducted in a biosafety level 3 facility.

### 2.4. Statistical Analysis

A statistical analysis was performed using a GraphPad PRISM 9.2.0 (GraphPad Software). For the purpose of statistical calculation, an MN titer of <10 was assigned a value of 5. A Wilcoxon matched-pairs signed rank test was used for comparing the MN titers against the wild type lineage A and lineage B.1.351 virus. The correlation between binding the antibody concentration (anti-RBD IgG or anti-N IgG) and the MN titer was determined using Spearman’s correlation. A *p* value of <0.05 was considered statistically significant.

## 3. Results

**Figure 1:**
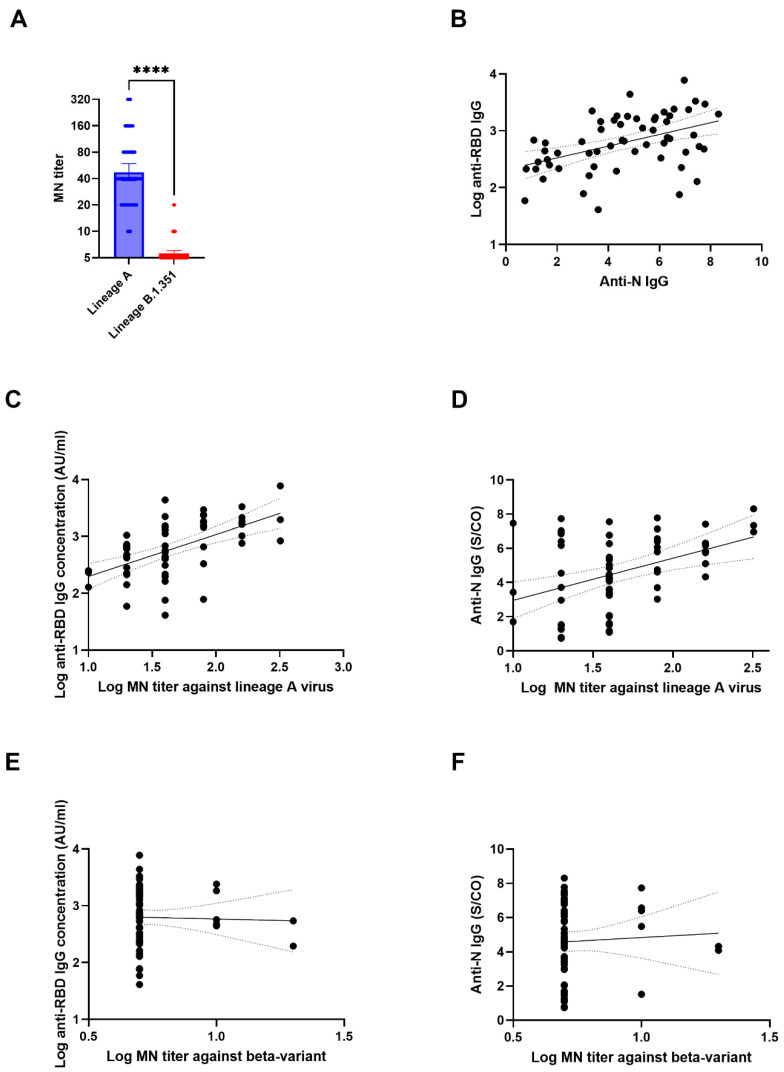
Correlation between MN titer and anti-N or anti-RBD IgG titers. (**A**) Comparison between MN titers against wild type lineage A virus (HKU-001a) and lineage B.1.351 virus (beta variant). The data represent geometric mean titer and 95% confidence interval ****, *p* < 0.0001. (**B**) Correlation between anti-RBD IgG and anti-N IgG. (**C**,**D**) Correlation between MN titer against wild type lineage A and anti-RBD IgG (**C**) or anti-N IgG (**D**); (**E**,**F**) Correlation between MN titer against lineage B.1.351 and anti-RBD IgG (**E**) or anti-N IgG (**F**). For (**B**–**F**), the solid lines indicate the best fit line, while the dotted lines indicate the 95% confidence interval.

The GMT against the wild type lineage A virus (GMT: 47.3; range: 10–320) was significantly higher than that against the beta variant (GMT: 5.6; range <10–20) (*p* < 0.0001) (Figure 1A). There was a statistically significant degree of correlation between the anti-RBD IgG and the anti-N IgG with a Spearman rho of 0.4666 (95% confidence interval [C.I. 0.2294–0.6514]; *p* = 0.0002) (Figure 1B).

There was a statistically significant correlation between the concentrations of the anti-RBD IgG or the anti-N IgG with the HKU-001a MN titer. The correlation between the anti-RBD IgG concentration and the MN titer (Spearman rho: 0.5901 [95% C.I. 0.3849–0.7398, P<0.0001]) was higher than that between the anti-N IgG S/CO and the MN titer (Spearman rho: 0.3827 [95% C.I. 0.1303–0.5884, *p* = 0.0030]) (Figure 1C,D). However, the correlation between the anti-RBD IgG or anti-N IgG with the MN titer against B.1.351 was not statistically significant (*p* = 0.8284 and *p* = 0.6938, respectively) (Figure 1E,F).

For specimens with anti-RBD IgG concentrations of ≥1000 AU/mL, 95.5% (21/22) had an MN titer against HKU-001A of ≥40. However, only 9.1% (2/22) had a beta variant MN titer of 10–20, and none had a beta variant titer of 40 or above (Table 1).

## 4. Discussion

SARS-CoV-2 binding antibody assays are usually used as a surrogate for determining the immune response because it is easy to perform without the need for a biosafety level 3 facility. Studies early during the COVID-19 pandemic showed that the anti-RBD IgG concentration has a high correlation with MN titers [13]. In this study, we confirmed that both the anti-RBD IgG and anti-N IgG concentrations showed a correlation with MN titers against the wild type lineage A virus. However, the anti-RBD IgG and anti-N IgG concentrations did not correlate with the MN titer against the beta variant. Even for serum with a high anti-RBD IgG ≥1000 AU/mL, 90.9% had no detectable MN antibody titer against the beta variant. This is of particular concern as our results suggest that the current commercial antibody binding assays that were designed prior to the appearance of RBD variants are not useful in predicting a neutralizing antibody response against some variants.

The beta variant contains the RBD mutations K417N, E484K, and N501Y. Since anti-RBD IgG measure all antibodies that bind to the RBD, our results suggest that these anti-RBD IgG do not neutralize the beta variant virus. Studies with recombinant RBD with different mutations showed that E484K alone can significantly affect the binding of serum antibodies to the RBD [10]. E484K has been shown to destabilize the native conformation at the tip of the RBD, which is important for neutralization [14].

There are several limitations in this study. First, we did not include pediatric patients in this study. Weisberg et al. showed the distinct antibody responses in children and adults after a SARS-CoV-2 infection. Adult patients generated antibodies against both the S and NP proteins, while children generated antibodies against the S protein [15]. Hence, the results may be different among children. Second, the anti-N assay is not intended to be a quantitative assay. Third, we have not tested the MN titer against other VOCs.

The commercial binding antibody assay, especially the anti-RBD IgG assays, are widely used for the assessment of vaccine immunity, especially in non-research settings. The results from this study suggest that the concentration of anti-RBD IgG as determined by these commercial antibody assays should not be used in predicting the level of neutralizing antibodies against variants. Newer versions of anti-RBD IgG incorporating RBD with different mutations would be required for a better correlation with neutralization antibody titers.

## Figures and Tables

**Table 1:** Correlation between binding antibody titer and microneutralization antibody titer.

MN Titer	Anti-RBD IgG (AU/mL)			Anti-N IgG (S/CO)		
	<50 (*n* = 1)	50–999 (*n* = 35)	≥1000 (*n* = 22)	<1.4 (*n* = 5)	1.4–4.9 (*n* = 26)	≥5.0 (*n* = 27)
Against wild type lineage A (HKU-001a)						
10–20	0 (0)	15 (42.9)	1 (4.5)	3 (60)	7 (26.9)	6 (22.2)
≥40	1 (100)	20 (57.1)	21 (95.5)	2 (40)	19 (73.1)	21 (77.8)
Against beta variant (B.1.351)						
<10	1 (100)	30 (85.7)	20 (90.9)	5 (100)	23 (88.5)	23 (85.2)
10–20	0 (0)	5 (14.3)	2 (9.1)	0 (0)	3 (11.5)	4 (14.8)
≥40	0 (0)	0 (0)	0 (0)	0 (0)	0 (0)	0 (0)

Data represent no. of patients (%).

## Data Availability

The data used to support the findings of this study are included within the article.
